# External validation of the GAP model in Chinese patients with idiopathic pulmonary fibrosis

**DOI:** 10.1111/crj.13564

**Published:** 2022-11-27

**Authors:** Xinran Zhang, Yanhong Ren, Bingbing Xie, Shiyao Wang, Jing Geng, Xuan He, Dingyuan Jiang, Jiarui He, Sa Luo, Xin Wang, Dingyun Song, Mingming Fan, Huaping Dai

**Affiliations:** ^1^ Department of Clinical research and Data management, Center of Respiratory Medicine, China‐Japan Friendship Hospital; National Center for Respiratory Medicine; Institute of Respiratory Medicine Chinese Academy of Medical Sciences; National Clinical Research Center for Respiratory Diseases Beijing China; ^2^ Department of Pulmonary and Critical Care Medicine, Center of Respiratory Medicine, China–Japan Friendship Hospital; National Center for Respiratory Medicine; National Clinical Research Center for Respiratory Diseases; Institute of Respiratory Medicine, Chinese Academy of Medical Science Peking Union Medical College Beijing China; ^3^ Beijing University of Chinese Medicine Beijing China; ^4^ The 2nd Hospital of Jilin University Changchun China

**Keywords:** external validation, GAP model, idiopathic pulmonary fibrosis, prognosis

## Abstract

**Introduction:**

The GAP model was widely used as a simple risk “screening” method for patients with idiopathic pulmonary fibrosis (IPF).

**Objectives:**

We sought to validate the GAP model in Chinese patients with IPF to evaluate whether it can accurately predict the risk for mortality.

**Methods:**

A total of 212 patients with IPF diagnosed at China‐Japan Friendship Hospital from 2015 to 2019 were enrolled. The latest follow‐up ended in September 2022. Cumulative mortality of each GAP stage was calculated and compared based on Fine‐Gray models for survival, and lung transplantation was treated as a competing risk. The performance of the model was evaluated in terms of both discrimination and calibration.

**Results:**

The cumulative mortality in patients with GAP stage III was significantly higher than that in those with GAP stage I or II (Gray's test *p* < 0.0001). The Harrell c‐index for the GAP calculator was 0.736 (95% CI: 0.667–0.864). The discrimination for the GAP staging system were similar with that for the GAP calculator. The GAP model overestimated the mortality rate at 1‐ and 2‐year in patients classified as GAP stage I (6.90% vs. 1.77% for 1‐year, 14.20% vs. 6.78% for 2‐year).

**Conclusions:**

Our findings indicated that the GAP model overestimated the mortality rate in mild group.

## Graphical abstract

The GAP model, which is widely used in grading patients with IPF, was externally validated in this study. After comprehensive analysis, the GAP model overestimated the mortality rate in a low‐risk group of Chinese patients with IPF. We modified the GAP model to make it more suitable in China.

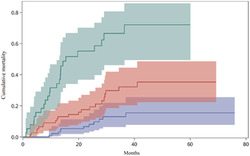

**List of Abbreviations**
CI: confidence interval
CPI: composite physiologic index
CRP: clinical‐radiologic‐physiologic
HR: hazard ratio
IIP: idiopathic interstitial pneumonias
IPF: idiopathic pulmonary fibrosis
UIP: usual interstitial pneumonia

## 1 INTRODUCTION

Idiopathic pulmonary fibrosis (IPF) is the most common and aggressive form of idiopathic interstitial pneumonias (IIP) with histopathological pattern being usual interstitial pneumonia (UIP).^1^ Mostly, IPF occurs in older adults and the lesion is limited to the lung. Reported incidence rates for IPF vary considerably depending on the method of data collection and diagnostic criteria.^2^, ^3^, ^4^, ^5^, ^6^ The incidence of IPF in North America and Europe was 2.8–9.3 per 100 000 per year, and the rates in Asia and South America were significantly lower.^7^ A population‐based study conducted in Taiwan found that the estimated incidence of IPF in Taiwan was 0.9–1.6 cases per 100 000 persons.^4^ Recently, the incidence of IPF is increasing worldwide.

The etiology of IPF is not clear. The progression of IPF reflected in inexorable decline in lung function and progressive respiratory failure. Mortality in patients with IPF is high, and once diagnosed, the median survival time will be 2–3 years.^8^ Thus, IPF has imposed a huge economic health care burden. However, there is considerable heterogeneity in disease progression among each patient. Most patients present with a slow and predictable decline in lung function, a few patients with repeated acute exacerbations, and a very small number of patients with a short period of progressive development. It is important for clinicians to accurately predict the prognosis of patients and make individualized treatment plans.

By now, several prognosis models have been developed to predict the risk of mortality in patients with IPF. For example, the clinical‐radiologic‐physiologic (CRP) scoring system established by King et al.,^9^ the composite physiologic index (CPI) established by Wells et al.^10^ and the GAP model established by Ley et al.^11^ Among them, the GAP model included four easily accessible predictors (gender, age, % predicted FVC and % predicted DL_CO_), and developed and internal validated using competing risk model. Due to the strengths above, the GAP model was widely used as a quick and simple screening method for estimating risk in patients with IPF and to estimate individual risk for certain patients. The GAP model was frequently used as it stands in China before.

In our present study, we aimed to assess the performance of the GAP model in predicting the risk of mortality in Chinese patients with IPF.

## 2 MATERIALS AND METHODS

### 2.1 Study population

Patients who diagnosed as IPF according to the American Thoracic Society/European Respiratory Society guideline and willing to participate in the study were consecutively enrolled between January 2015 and December 2019 in China‐Japan Hospital. Patients who had not undergone pulmonary function testing (PFT) that included DL_CO_ measurement were excluded from the study, as well as those without result of follow‐up. Finally, a total of 212 patients were included in the study. The follow‐up ended in September 2022. The present study was approved by the Ethic Committee of China‐Japan Hospital.

### 2.2 Data collection and follow‐up assessment

During the study, baseline clinical parameters of patients in hospitalization were collected. We collected their demographic information, such as age, gender, and smoking status. Smoking status was categorized as never, current and former smoking. Comorbidities were classified into different categories according to the Charlson comorbidity index suggested by Charlson et al.,^12^ and the Charlson comorbidity index was further calculated. Baseline spirometry data like percent predicted forced vital capacity (FVC, % predicted), percent predicted diffusing capacity of the lungs for carbon monoxide (DL_CO_, % predicted), percent predicted forced expiratory volume in 1 s (FEV1, % predicted) and FEV1/FVC was also collected. Survival information was obtained by retrieving medical records and telephone interviews, including survival status, cause of death, time of death, whether or not has received lung transplantation and time of lung transplantation.

### 2.3 The GAP model

In 2012, the GAP model was suggested by Ley et al.^11^ and calculated using the following predictors: gender, age, FVC (% predicted) and DL_CO_ (% predicted). The GAP model included the GAP calculator, GAP index and GAP staging system. The algorithm to calculate mortality predicted by the GAP calculator was shown in Supporting Information. The GAP index was calculated according to the points shown in Table S1 and then used to classify patients into stage I (0–3 points), stage II (4–5 points) or stage III (6–8 points). The mortality risk predicted by the GAP stage was shown in Table S2. Patients classified as GAP stage I have the lowest risk for mortality and may not require immediate listing for lung transplantation. Those classified as GAP stage III have the highest risk for mortality, and palliative care would be referred if not a transplant candidate.

### 2.4 Statistical analysis

Continuous variables were expressed as median (interquartile range) and tested by the Kruskal–Wallis test. Categorical variables were expressed as number (percentage) and tested by the chi‐square test or Fisher's exact test. Cumulative mortality of each GAP stage was calculated and compared based on Fine–Gray models for survival, and lung transplantation was treated as a competing risk. Hazard ratio and 95% confidence interval (CI) were calculated using competing risk regression model. The performance of the model was evaluated in terms of both discrimination and calibration. Harrell c‐index was used to evaluate discrimination of GAP calculator and staging system. Calibration plots were used to compare the predicted risk of death by the GAP calculator and staging system with the observed mortality. Statistical analyses were performed using SAS software, version 9.4 (SAS Institute Inc.) and R software (version 3.6.1).

## 3 RESULTS

### 3.1 Baseline characteristics

A total of 212 patients with IPF were assessable and included in the current analysis, and the baseline characteristics were shown in Table 1. According to the GAP staging system, 115 (54.25%) patients were classified as GAP stage I, 76 (35.85%) patients were classified as GAP stage II, and 21 (9.90%) patients were classified as GAP stage III. Age, gender, smoking status, FEV1 (% predicted), FVC (% predicted), FEV1/FVC (%) and DL_CO_ (% predicted) showed significant differences between GAP stages (all *p* < 0.05). Age, the percentage of males, the percentage of former smoker and FEV1/FVC (%) increased with the increase of GAP stage, whereas FEV1 (% predicted), FVC (% predicted) and DL_CO_ (% predicted) decreased with the increase of GAP stage (all *p* < 0.05). There was no significant difference in comorbidities. The most common comorbidities included mild liver disease (29.25%), chronic pulmonary disease (28.77%) and diabetes mellitus (27.83%) after classification according to Charlson comorbidity index.

**TABLE 1:** The baseline characteristics of patients with IPF stratified by GAP stages

Characteristics	Total (*n* = 212)	Stage I (*n* = 115)	Stage II (*n* = 76)	Stage III (*n* = 21)	*p* value
Age (years)	65.00 (60.00, 71.00)	62.00 (59.00, 68.00)	67.50 (63.00, 72.00)	70.00 (68.00, 73.00)	<0.0001
Males	177 (83.49)	87 (75.65)	70 (92.11)	20 (95.24)	0.002
Smoking					
Nonsmoker	53 (25.00)	34 (29.57)	16 (21.05)	3 (14.29)	0.022
Current smoker	34 (16.04)	24 (20.87)	9 (11.84)	1 (4.76)	
Former smoker	125 (58.96)	57 (49.57)	51 (67.11)	17 (80.95)	
Smoking duration (years)	30.00 (25.00, 40.00)	30.00 (26.50, 40.00)	30.00 (20.00, 40.00)	40.00 (30.00, 40.00)	0.527
Smoking exposure (pack‐years)	33.00 (20.00, 45.00)	30.00 (20.00, 42.50)	40.00 (20.00, 50.00)	27.50 (15.00, 50.00)	0.521
FEV1, % predicted	81.90 (67.75, 95.05)	89.10 (79.70, 101.30)	71.05 (61.20, 83.40)	64.10 (50.30, 78.40)	<0.0001
FVC, % predicted	79.85 (64.35, 94.55)	91.80 (79.80, 101.90)	68.75 (56.35, 80.30)	59.70 (46.90, 66.60)	<0.0001
FEV1/FVC, %	81.65 (76.54, 86.18)	79.98 (75.76, 85.51)	82.50 (78.76, 87.34)	86.70 (82.58, 92.14)	0.001
DL_CO_, % predicted	50.75 (37.20, 63.65)	60.90 (51.20, 68.90)	38.65 (31.50, 49.20)	30.20 (24.60, 33.90)	<0.0001
Charlson Comorbidity index	1.00 (0.00, 2.00)	1.00 (1.00, 2.00)	1.00 (0.00, 2.00)	1.00 (0.00, 2.00)	0.242
Comorbidity					
Myocardial infarction	7 (3.30)	2 (1.74)	4 (5.26)	1 (4.76)	0.283
Congestive heart failure	5 (2.36)	2 (1.74)	1 (1.32)	2 (9.52)	0.091
Peripheral vascular disease	16 (7.55)	8 (6.96)	7 (9.21)	1 (4.76)	0.738
Cerebrovascular disease	24 (11.32)	14 (12.17)	9 (11.84)	1 (4.76)	0.542
Dementia	0	0	0	0	/
Chronic pulmonary disease	61 (28.77)	32 (27.83)	22 (28.95)	7 (33.33)	0.876
Rheumatic disease	0	0	0	0	/
Peptic ulcer disease	4 (1.89)	3 (2.61)	0	1 (4.76)	0.147
Mild liver disease	62 (29.25)	40 (34.78)	16 (21.05)	6 (28.57)	0.124
Diabetes without chronic complication	59 (27.83)	34 (29.57)	21 (27.63)	4 (19.05)	0.613
Diabetes with chronic complication	1 (0.47)	0	1 (1.32)	0	0.458
Hemiplegia or paraplegia	0	0	0	0	/
Renal disease	3 (1.42)	2 (1.74)	0	1 (4.76)	0.198
Any malignancy	13 (6.13)	9 (7.83)	4 (5.26)	0	0.193
Moderate or severe liver disease	0	0	0	0	/
Metastatic solid tumor	4 (1.89)	4 (3.48)	0	0	0.316
AIDS/HIV	0	0	0	0	/

*Note*: Data were expressed as median (interquartile range) or number (percentage) where appropriate.

^*^ *p* value was calculated using the Kruskal–Wallis test or the chi‐square test, Fisher exact test when appropriate.

### 3.2 Survival analysis

The median follow‐up duration of the study population was 26.72 months (range: 1.03 months to 75.87 months). At the end of follow‐up, 58 patients died and 14 patients had received lung transplantation. Of those who died, 49 patients (84.48%) died from respiratory failure and nine patients (15.52%) from other causes. The observed cumulative mortality rate at 1, 2 and 3 years were 10.48%, 20.56% and 29.68%, respectively.

**FIGURE 1:**
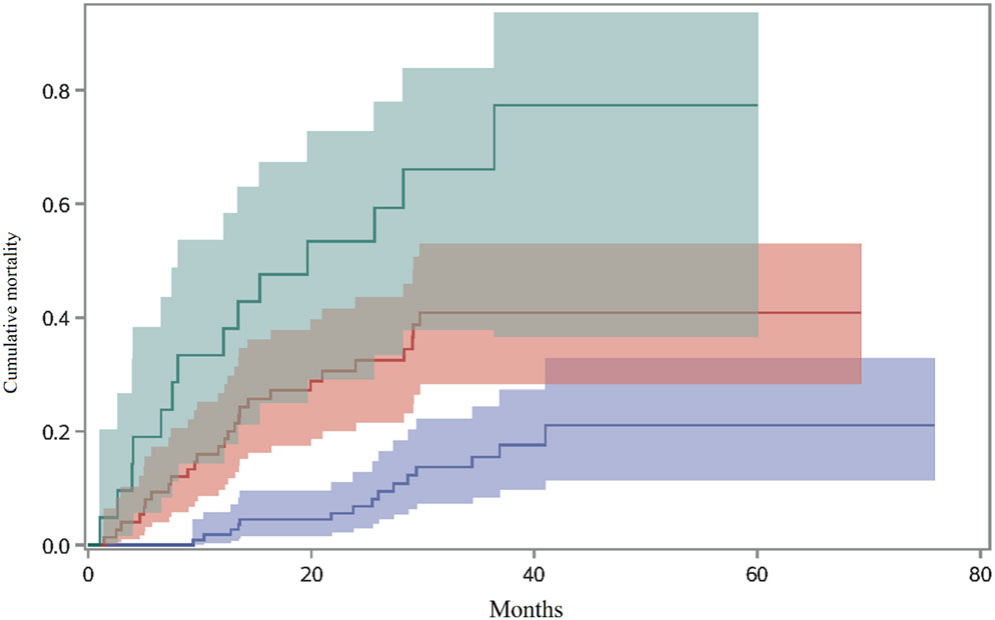
The cumulative mortality of patients with IPF by the GAP staging system. Blue represents patient with GAP stage I. Red represents patient with GAP stage II. Green represents patient with GAP stage III.

As shown in Figure 1, the cumulative mortality increased significantly with the increase of GAP stage, and the cumulative mortality in patients with GAP stage III was significantly higher than that in those with GAP stage I or II (Gray's test *p* < 0.0001). Survival analysis of GAP calculator components with competing risk model was conducted, and the results were shown in Table 2. Univariate analysis found that the risk of death significantly decreased as FVC (% predicted, HR: 0.97, 95% CI: 0.96–0.98) and DL_CO_ (% predicted, HR: 0.96, 95% CI: 0.95–0.98) increased. After multivariate analysis, age, FVC (% predicted) and DL_CO_ (% predicted) showed significant association with mortality, the HRs (95% CI) were 1.05 (1.01–1.09), 0.98 (0.96–1.00) and 0.97 (0.94–0.99), respectively.

**TABLE 2:** Survival analysis of GAP calculator components with competing risk model

Characteristics	Univariate		Multivariate	
	HR (95% CI)	*p* value	HR (95% CI)	*p* value
Age	1.05 (1.01–1.08)	0.011	1.05 (1.01–1.09)	0.029
Gender				
Female	Reference		Reference	
Male	1.02 (0.51–2.01)	0.962	1.85 (0.65–5.30)	0.250
FVC, % predicted	0.97 (0.96–0.98)	<0.0001	0.98 (0.96–1.00)	0.026
DL_CO_, % predicted	0.96 (0.95–0.98)	<0.0001	0.97 (0.94–0.99)	0.010
Image type				
Non‐UIP	Reference		Reference	
UIP	0.91 (0.51–1.63)	0.756	0.76 (0.42–1.37)	0.357
Smoking				
Nonsmoker	Reference		Reference	
Current smoker	0.76 (0.31–1.86)	0.541	1.33 (0.44–3.99)	0.614
Former smoker	1.22 (0.64–2.33)	0.549	1.21 (0.55–2.64)	0.632
Fibrosis score	1.35 (0.98–1.86)	0.063	0.93 (0.61–1.41)	0.729
Pulmonary comorbidity	1.47 (0.85–2.52)	0.167	1.77 (0.93–3.37)	0.083

*Note*: Multivariate model was adjusted for age, gender, FVC (% predicted) and DL_CO_ (% predicted).

The association between smoking, image type, fibrosis score, pulmonary comorbidity and death risk was also explored. However, after multivariate analysis, no significant association was found (Table 2).

### 3.3 External validation of the GAP model

We evaluated the model performance by calibration and discrimination. The Harrell c‐index for the GAP calculator was 0.736 (95% CI: 0.667–0.864). The discrimination for the GAP staging system were similar with that for the GAP calculator, with c‐index being 0.721 (95% CI: 0.663–0.778).

**FIGURE 2:**
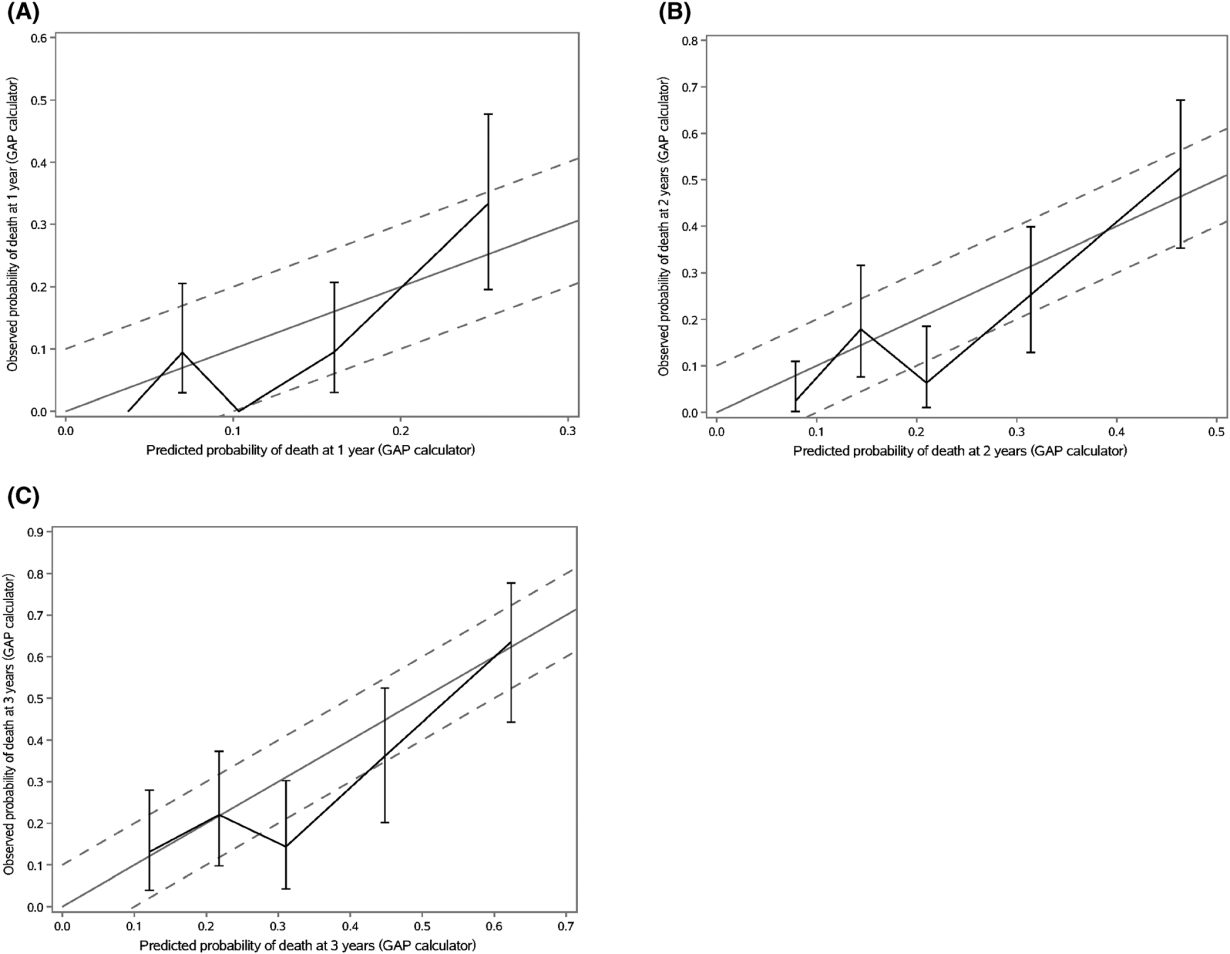
Calibration plots of the GAP calculator in patients with IPF. The x‐axis shows the predicted probability of death by the GAP calculator, and the y‐axis shows the observed probability of death. The solid line represents perfect agreement between predicted and observed risks, and the dashed line represents ±10% differences between them.

**FIGURE 3:**
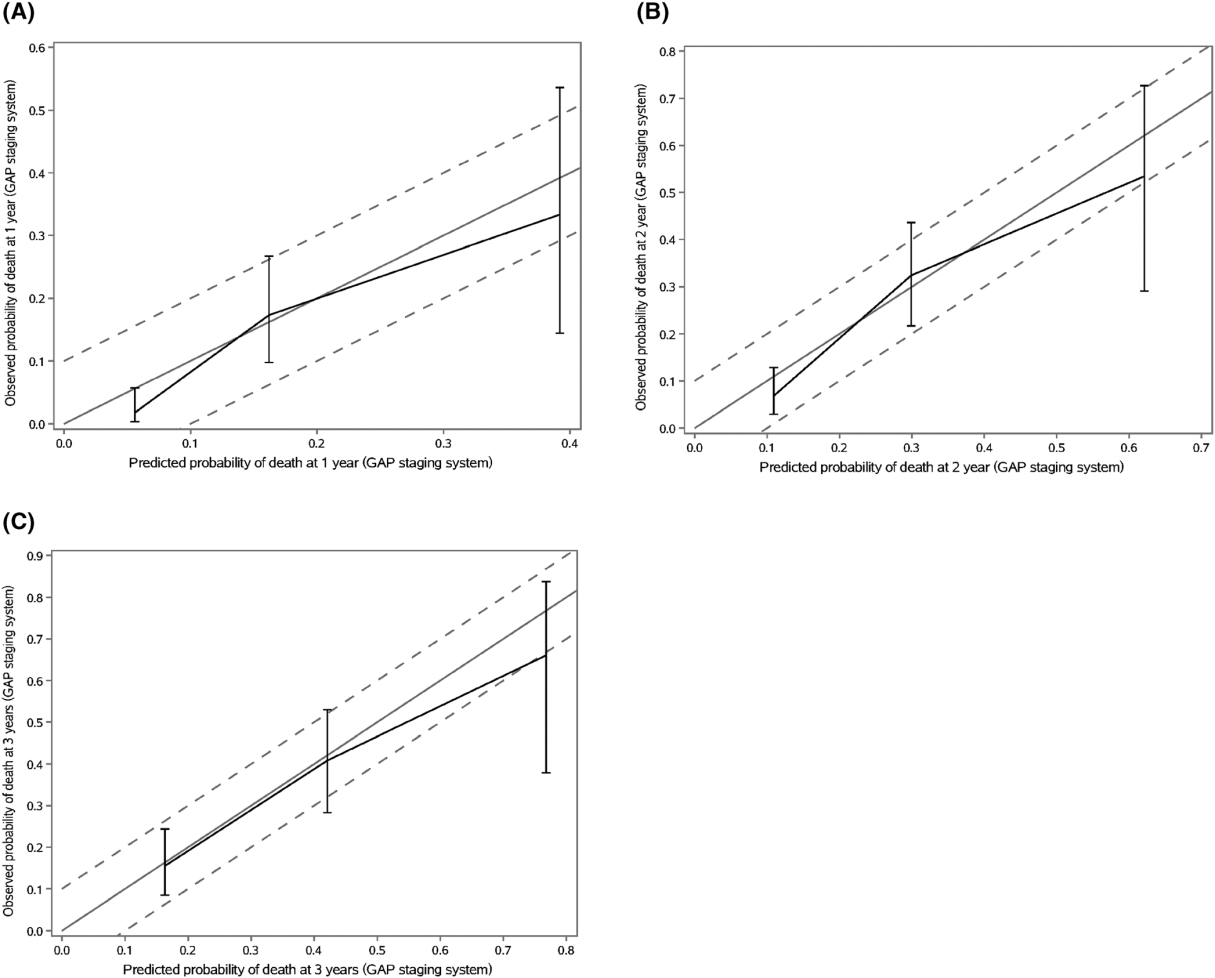
Calibration plots of the GAP staging system in patients with IPF. The x‐axis shows the predicted probability of death by the GAP staging system, and the y‐axis shows the observed probability of death. The solid line represents perfect agreement between predicted and observed risks, and the dashed line represents±10% differences between them.

Table 3 showed the predicted and observed mortality rate in each GAP stage. The observed risks of 1y‐ and 2y‐mortality in patients classified as GAP stage I were 1.77% and 6.78%, respectively, which were apparently lower than the predicted risk of death by GAP calculator (1y‐mortality: 6.90%, 2y‐mortality: 14.20%) and GAP staging system (1y‐mortality: 5.60%, 2y‐mortality: 10.90%) in patients that classified as GAP stage I, whereas the differences in patients with GAP stage II and III were not significant. The calibration plots demonstrated that the GAP calculator overestimated the risk for 1y‐, 2y‐ and 3y‐mortality, whereas the GAP staging system predicted mortality better than the GAP calculator (Figures 2 and 3).

**TABLE 3:** Comparison of predicted and observed cumulative mortality

GAP stage	Death (no.)	Observed cumulative mortality (%)	Predicted mortality by GAP calculator (%)	Predicted mortality by GAP stage (%)
1‐year mortality	22	10.48	12.45	
Stage I	1	1.77	6.90	5.60
Stage II	14	17.32	16.49	16.20
Stage III	7	33.33	28.23	39.20
2‐year mortality	39	20.56	24.20	
Stage I	6	6.78	14.20	10.90
Stage II	23	32.42	31.95	29.90
Stage III	10	53.44	50.91	62.10
3‐year mortality	46	28.78	34.41	
Stage I	9	15.48	21.30	16.30
Stage II	26	40.85	45.17	42.10
Stage III	11	66.05	67.26	76.80

### 3.4 Modify the GAP stage system

Since gender was not associated with mortality risk, we modified the GAP stage system by excluding gender from the indicators. We also reassigned values for age, FVC (% predicted) and DL_CO_ (% predicted). The points for each predictor were shown in Table 4, and the total points ranged from 0 to 9. The reassigned points were significantly associated with the death risk (HR: 1.36, 95% CI: 1.23–1.51).

**TABLE 4:** Modified points for predictors and mortality for modified GAP stage

Predictors	Modified points
Age, year	
≤60	0
61–65	1
>65	3
FVC, % predicted	
>75	0
≤75	3
DL_CO_, % predicted	
>55	0
36–55	1
≤35	3
Total possible points	9

Modified GAP stage			
Stage	I	II	III
Points	0–3	4–6	7–9
1‐y	2.13	10.39	31.58
2‐y	8.15	19.35	55.14
3‐y	13.04	32.13	66.54

**FIGURE 4:**
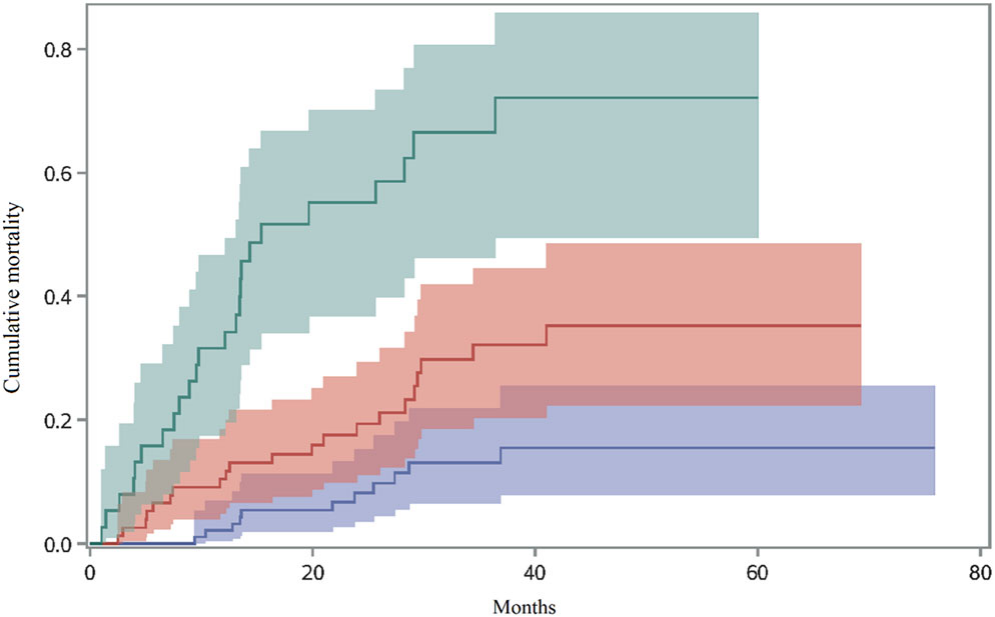
The cumulative mortality of patients with IPF by the modified GAP staging system. Blue represents patient with the modified GAP stage I. Red represents patient with the modified GAP stage II. Green represents patient with the modified GAP stage III.

We divided the patients into three groups according to their modified points. Patients with points less than 4 points were classified into modified GAP stage I, patients with points between 4 and 6 were modified GAP stage II, whereas patients with points more than 6 were modified GAP stage III. As shown in Figure 4, the cumulative mortality increases by the modified GAP stage, and the cumulative mortalities for modified GAP stages I, II and III were 15.46%, 35.31% and 72.12%, respectively (Gray's test *p* < 0.0001). Comparing with modified GAP stage I, the mortality risk for patients with modified GAP stage II (HR: 2.58, 95% CI: 1.26–5.28) and stage III (HR: 8.93, 95% CI: 4.36–18.27) significantly increased.

## 4 DISCUSSION

In the present study, we evaluated the model performance of the GAP model in predicting the mortality risk in a cohort of Chinese patients with IPF. We found that the calibration and discrimination of the GAP model in Chinese patients with IPF were not as well as that in Western population. The GAP model overestimated the mortality rate at 1 and 2 years in patients classified as GAP stage I.

In previous studies, several predictive models have been established to predict the prognosis of patients with IPF. The predictive model of prognosis is to predict the risk of mortality in the future by combining multiple predictive indicators of diseases. In 2001, King et al.^9^ established the CRP scoring system based on the results of age, smoking history, clubbing, extent of profusion of interstitial opacities and presence or absence of pulmonary hypertension on the chest radiograph, TLC (% predicted), and PaO2 at the end of maximal exercise. In 2003, Wells et al.^10^ established the CPI which is a combination of FVC (% predicted), FEV1 (% predicted) and DL_CO_ (% predicted), to reflect the morphologic extent of pulmonary fibrosis. The CPI correlated with the extent of pulmonary fibrosis on CT more strongly, and could reflect the severity of pulmonary fibrosis disease without the confounding effect of emphysema. In 2011, du Bois et al.^13^ established a predictive model considering the changes of predictors during follow‐up. The complete model included age, respiratory hospitalization, FVC (% predicted), 24‐week change in FVC, DL_CO_ (% predicted), 24‐week change in DL_CO_ (% predicted), and 24‐week change in health‐related quality of life. In 2012, Ley et al.^11^ established the GAP model based on gender, age, and lung physiology. The GAP model included the GAP calculator which was a continuous model, GAP index and GAP staging system. The predictors included in the GAP model were easy to obtain and can be used to measure the patient's comprehensive condition from the clinical and physiological dimensions. Among these models, the GAP model was the simplest clinical prediction model, and has performed internal validation. Thus, the GAP model, especially GAP staging system, was widely used to grade the severity of patients with IPF worldwide, as well as in China.

The GAP model was developed and validated in Western population, who were different from Asian population in both environmental and genetic factors. Several studies have evaluated the performance of the GAP model in Korea and Japan. In 2015, Kim et al.^14^ validated the GAP model in 268 patients with IPF diagnosed from 2005 to 2009 in Korea, and found that the discrimination power was similar to the original study, but did not predict the 3‐year risk of death accurately. In 2016, Kondoh et al.^15^ evaluated the performance of the GAP model in 326 patients with IPF diagnosed from 2003 to 2007 in Japan. The survival between Japanese patients with GAP stage II and III was not significantly different, and the mortality rates in patients with GAP stage I and II were underestimated.

So far, there was no predictive model to evaluate the prognosis of Chinese patients with IPF. The GAP model was used widely to grade the severity of patients in clinical practice in China. Nevertheless, whether the GAP model was applicable to Chinese population needs to be verified. Chen et al.^16^ evaluated the prognostic effect of GAP stage and CPI in Chinese IPF patients and found that there was no statistical difference in 1‐year survival rate between different GAP stages. However, Cai et al.^17^ found that GAP staging system showed a strong ability to predict the risk of death in IPF patients evaluated by AUC curve. CPI was significantly better than GAP staging in the prediction of overall mortality, 2‐year mortality and 3‐year mortality. Zhuang et al.^18^ found that the 3‐year and 5‐year mortality of GAP stage III significantly inferior than that of stage I and II. In our current study, the discrimination and calibration of the GAP model were comprehensively evaluated in Chinese patients with IPF. We found that the GAP model did not work so well for the Chinese population as for Western population. Discrimination measured whether the GAP model can discriminate the patients with death from those without. A well‐performed model can spread the risk scores of patients with death as far as possible from those without. However, the discrimination ability of the GAP calculator and GAP stage was not good with the c‐index being 0.736 and 0.721, respectively. Calibration measured the accuracy of absolute mortality risk predicted by the GAP model, that was, whether the mortality predicted by the GAP model was consistent with the mortality observed. Unfortunately, the GAP model overestimated the mortality rate at 1y‐ and 2y‐mortality in patients classified as GAP stage I. The predicted mortality were 3.2 and 1.6 times the observed mortality.

Although the performance of the GAP model was not satisfactory in Chinese patients, we found that the prognosis of patients with GAP stage III were significantly worse than patients with GAP stage I or stage II. This result suggested that the GAP model was associated with prognosis. We have evaluated the prognosis effect of the four predictors used in the GAP model and found that mortality was significantly associated with age, FVC (% predicted) and DL_CO_ (% predicted). Gender was not associated with prognosis in our present study, possibly because of the high percentage of males in our cohort. The percentage of males in patients with GAP stage II and III was more than 90%. Based on the above results, we modified the GAP stage by excluding gender, and found that the survival of patients with modified stage III was significantly worse.

Compared with the derivation cohort of the GAP model, our cohort showed better baseline spirometry result and higher percentage of males. The proportion of comorbidities was low in our cohort, and comorbidities, especially emphysema and pulmonary arterial hypertension, have significant impact on prognosis. Meanwhile, with the improvement of diagnostic criteria and the promotion of doctor's awareness, patients with IPF were identified earlier and more frequently.^19^ The median survival time for patients with IPF aged 65 years and older were longer in 2011 than they were 10 years before in United States (4.0 years in 2011 vs. 3.3 years in 2007).^3^ In China, patients with IPF were getting more and more standardized treatment, which has reduced the mortality risk. Our previous study demonstrated that 1‐, 2‐ and 5‐year mortality rate for Chinese patients with IPF from 1999 to 2007 were 39%, 48%, and 61%, respectively.^8^ In our current study, patients were enrolled from 2015 to 2019, and 1‐, 2‐ and 3‐year cumulative mortality rate were 10.48%, 20.56% and 29.68%, respectively. All of our enrolled patients were followed up for more than 2 year, and the 1‐year mortality for mild patients with IPF in our present study was even less than 2%. These results suggested that the short‐term prognosis for patients with IPF was improving in China.

There were several potential limitations in this study. First, this study was carried out in one tertiary referral hospital, which restricted the generalizability in the whole Chinese population. Second, to be consistent with the method of model development and validation, all‐cause of mortality was analyzed in this study. Third, patients without the result of DL_CO_ were excluded from this study, since we could not confirm why DL_CO_ examination was not performed from medical record. Fourth, the follow‐up period was not long enough to reach the median survival time, so we only evaluated the predict effect for short‐term survival. The long‐term survival will be evaluated in the future.

## 5 CONCLUSION

In conclusion, our findings indicated that the GAP model showed poor discriminative ability and overestimated the mortality in patients classified as GAP stage I in Chinese population. Thus, the GAP model was not suitable to Chinese population and was not appropriate for grading patient's severity. There is an urgent need to develop methods for assessing the prognostic risk by multicenter studies in Chinese population with IPF in the future.

## CONFLICT OF INTEREST

No potential conflicts of interest exist with any companies/organizations whose products or services may be discussed in this article.

## ETHICS STATEMENT

This study protocol was reviewed and approved by the Ethic Committee of China‐Japan Hospital, approval number: 2017‐25‐1. Written informed consent was obtained from participants.

## AUTHOR CONTRIBUTION

XZ contributed to the design and statistical analyses of the study, and the writing and revision of the manuscript. YR contributed to the implementation of clinical study, data collection and analysis. HD contributed to the conception and design of the study, the revision of the manuscript, and the final approval of the version to be published. The other authors collected the data for this analysis. All authors have read and approved the final manuscript prior to submission.

## Supporting information


**Table S1** Calculation method of GAP score
**Table S2** Mortality risk predicted by the GAP stage

## Data Availability

The datasets used and analyzed during the current study are available from the corresponding author on reasonable request.
