# Supplementary material for: External validation of the GAP model in Chinese patients with idiopathic pulmonary fibrosis
Source: Clin Respir J. 2022 Nov 27;17(9):831–40. doi: 10.1111/crj.13564 (PMC10500316; doi:10.1111/crj.13564)
Supplement: Supplementary file 1 — Table S1 Calculation method of GAP score Table S2 Mortality risk predicted by the GAP stage [file CRJ-17-831-s001.docx]

Calculating mortality risks by using the GAP calculator

Step 1: Calculate S

S = [0.337 (GENDER) - 0.015 (FVC-68.464) + 0.092 (AGE1-67.676) -0.052 (AGE2) +

2.237 (DLCO1) + 0.024 (DLCO2)] x 0.909

Where:

1. GENDER =
2. 0.293 if the patient is male
3. -0.707 if the patient is female
4. FVC = forced vital capacity, % predicted
5. AGE1 = patient’s age, y
6. AGE2 = refer to the table below. Enter the value of AGE2 that corresponds to the patient’s age.

| AGE | AGE2 | AGE | AGE2 | AGE | AGE2 | AGE | AGE2 |
| --- | --- | --- | --- | --- | --- | --- | --- |
| ≤50 | 0 | 60 | 0.236 | 70 | 6.345 | 80 | 22.043 |
| 51 | 0 | 61 | 0.408 | 71 | 7.625 | 81 | 23.739 |
| 52 | 0 | 62 | 0.648 | 72 | 9.009 | 82 | 25.435 |
| 53 | 0 | 63 | 0.968 | 73 | 10.481 | 83 | 27.130 |
| 54 | 0 | 64 | 1.378 | 74 | 12.027 | 84 | 28.826 |
| 55 | 0 | 65 | 1.890 | 75 | 13.632 | 85 | 30.522 |
| 56 | 0.002 | 66 | 2.516 | 76 | 15.280 | 86 | 32.217 |
| 57 | 0.015 | 67 | 3.266 | 77 | 16.959 | 87 | 33.913 |
| 58 | 0.051 | 68 | 4.153 | 78 | 18.652 | 88 | 35.609 |
| 59 | 0.121 | 69 | 5.183 | 79 | 20.348 | 89 | 37.301 |

1. DLCO1=
2. 0.921 if the patient could not do the DLCO test
3. -0.079 if the patient could not do the DLCO test
4. DLCO2=
5. -50.549 if the patient could not do the DLCO test
6. (49.451 – the patient’s DLCO) if the patient could do the test

Step 2: Calculate risk using S:

1-y risk = 100 x [1-exp (-exp (S) x 0.225)]

2-y risk = 100 x [1-exp (-exp (S) x 0.486)]

3-y risk = 100 x [1-exp (-exp (S) x 0.768)]

Table S1 Calculation method of GAP score

| Predictors | Points |
| --- | --- |
| Gender |  |
| Female | 0 |
| Male | 1 |
| Age, yr |  |
| ≤60 | 0 |
| 61-65 | 1 |
| >65 | 2 |
| Physiology |  |
| FVC, % predicted |  |
| >75 | 0 |
| 50-75 | 1 |
| <50 | 2 |
| DLCO, % predicted |  |
| >55 | 0 |
| 36-55 | 1 |
| ≤35 | 2 |
| Can not perform | 3 |
| Total possible points | 8 |

Table S2 Mortality risk predicted by the GAP stage

| Stage | I | II | III |
| --- | --- | --- | --- |
| Points | 0-3 | 4-5 | 6-8 |
| 1-y | 5.6 | 16.2 | 39.2 |
| 2-y | 10.9 | 29.9 | 62.1 |
| 3-y | 16.3 | 42.1 | 76.8 |
